# Smart Pharmaceutical Monitoring System With Personalized Medication Schedules and Self-Management Programs for Patients With Diabetes: Development and Evaluation Study

**DOI:** 10.2196/56737

**Published:** 2025-02-11

**Authors:** Jian Xiao, Mengyao Li, Ruwen Cai, Hangxing Huang, Huimin Yu, Ling Huang, Jingyang Li, Ting Yu, Jiani Zhang, Shuqiao Cheng

**Affiliations:** 1 Department of Pharmacy Xiangya Hospital Central South University Changsha China; 2 The Hunan Institute of Pharmacy Practice and Clinical Research Changsha China; 3 Department of Pharmacy Dali University Dali China; 4 School of Medicine Wuhan University of Science and Technology Wuhan China; 5 Department of Senile Endocrinology Xiangya Hospital Central South University Changsha China

**Keywords:** pharmaceutical services, diabetes, self-management, intelligent medication scheduling system, drug database, GPT-4

## Abstract

**Background:**

With the climbing incidence of type 2 diabetes, the health care system is under pressure to manage patients with this condition properly. Particularly, pharmacological therapy constitutes the most fundamental means of controlling blood glucose levels and preventing the progression of complications. However, its effectiveness is often hindered by factors such as treatment complexity, polypharmacy, and poor patient adherence. As new technologies, artificial intelligence and digital technologies are covering all aspects of the medical and health care field, but their application and evaluation in the domain of diabetes research remain limited.

**Objective:**

This study aims to develop and establish a stand-alone diabetes management service system designed to enhance self-management support for patients, as well as to assess its performance with experienced health care professionals.

**Methods:**

Diabetes Universal Medication Schedule (DUMS) system is grounded in official medicine instructions and evidence-based data to establish medication constraints and drug-drug interaction profiles. Individualized medication schedules and self-management programs were generated based on patient-specific conditions and needs, using an app framework to build patient-side contact pathways. The system’s ability to provide medication guidance and health management was assessed by senior health care professionals using a 5-point Likert scale across 3 groups: outputs generated by the system (DUMS group), outputs refined by pharmacists (intervention group), and outputs generated by ChatGPT-4 (GPT-4 group).

**Results:**

We constructed a cloud-based drug information management system loaded with 475 diabetes treatment–related medications; 684 medication constraints; and 12,351 drug-drug interactions and theoretical supports. The generated personalized medication plan and self-management program included recommended dosing times, disease education, dietary considerations, and lifestyle recommendations to help patients with diabetes achieve correct medication use and active disease management. Reliability analysis demonstrated that the DUMS group outperformed the GPT-4 group in medication schedule accuracy and safety, as well as comprehensiveness and richness of the self-management program (*P*<.001). The intervention group outperformed the DUMS and GPT-4 groups on all indicator scores.

**Conclusions:**

DUMS’s treatment monitoring service can provide reliable self-management support for patients with diabetes. ChatGPT-4, powered by artificial intelligence, can act as a collaborative assistant to health care professionals in clinical contexts, although its performance still requires further training and optimization.

## Introduction

Chronic diseases are prevalent among the older adult population [1]. Diabetes, a common chronic disease, imposes a significant self-management burden on individuals and their families. Individuals with type 2 diabetes are increasingly being treated with multiple-medication regimens, including oral medications and/or exogenous insulin. Long-term medication and lifestyle changes are necessary for the successful management of both type 1 and type 2 diabetes [2]. However, the low success rate of diabetes self-management reflects some of the burdensome drug regimens and side effects [3]. A study pointed out that approximately 43.4% of patients with diabetes in low- and middle-income countries fail to adhere to their prescribed treatments, resulting in adverse health outcomes [4,5]. National survey data from the United States report basic knowledge gaps for many adults with diabetes, wherein only less than 50% are aware of their glycemic control levels. Guidelines emphasize that all patients should receive diabetes self-management education and support (DSMES), including regular medication taking, self-monitoring their blood glucose, changes in diet and physical exercise, foot self-care, and routine check-ups [2,6,7].

Nowadays, many apps and smart devices are being developed to enable patients to take their medications correctly and on time [8-13]. Some types of medicine boxes have also been proposed, but due to the complexity of filling, large volume, and lack of portability, most are not suitable for older adults [8,11,14]. In addition, producing the medicine box incurs certain costs, and many devices lack personalized medication guidance. Gupta et al [15] integrated drug names and doctor-prescribed schedules into a hardware device to provide reminders, but it lacked a friendly interface for interaction with health care professionals. Similarly, Wachira et al [16] designed and developed a guideline-encoding and execution engine using the JSON format and an optimization algorithm for medication plans through computer-aided output, yet their approach did not account for potential drug-drug interactions (DDIs) or real-world clinical data validation. In a report on the treatment plans and health status of individuals with diabetes, possible interactions were identified in 413 cases, and 53.1%, 7.8%, and 7.2% of the participants presented with moderate, minor, and serious risk of DDIs, respectively. Older adult patients are the primary users of multiple medications and consequently are more susceptible to inappropriate use, polypharmacy, and DDIs [17].

It is worth noting that generative artificial intelligence (AI)—based large language models (LLMs) have demonstrated significant potential innovation in health care in recent years. One such advanced model is ChatGPT [18], developed by OpenAI, which is currently among the largest publicly available language models [19]. ChatGPT-4 excels in a variety of medical evaluation benchmarks such as the United States Medical Licensing Examination (USMLE) [20,21] owing to its strong logical self-consistency and data analysis capabilities [22]. These LLMs leverage chat-based interfaces to respond to complex queries and solve problems [23]. Incorporating AI into the health care systems has the potential to significantly reduce task completion times and optimize workflows, thereby improving overall industry efficiency. As the availability and accessibility of these models increase, understanding their potential and limitations in real-world applications is essential. Song et al [24] evaluated ChatGPT-4’ s ability to provide health education to patients with urolithiasis across two clinical scenarios involving 21 questions. While the model had high accuracy, its responses lacked contextual relevance in some cases. However, the performance of generative AI in DSMES remains an area that requires further exploration and evaluation.

The study aims to develop a medication reminder system tailored for older patients with diabetes. Additionally, to explore the real-world application of this system, we compared its capabilities in providing patient medication plans and self-management programs with those of the latest publicly available version of ChatGPT. It provides reference for the auxiliary decision-making function and the ability to empower the pharmaceutical field of generative AI in medicine.

## Methods

### Infrastructure Work

#### Pharmaceutical Constraint Management

Building upon the findings from our team’s earlier work [25], we developed the Diabetes Universal Medication Schedule (DUMS) system as a more comprehensive solution for diabetes management. The system was built using the Python web framework, Flask, and the Flutter framework, written in Dart and Visual Studio Code. Data management and storage were implemented using a MySQL database and a Redis cache database.

**Figure 1:**
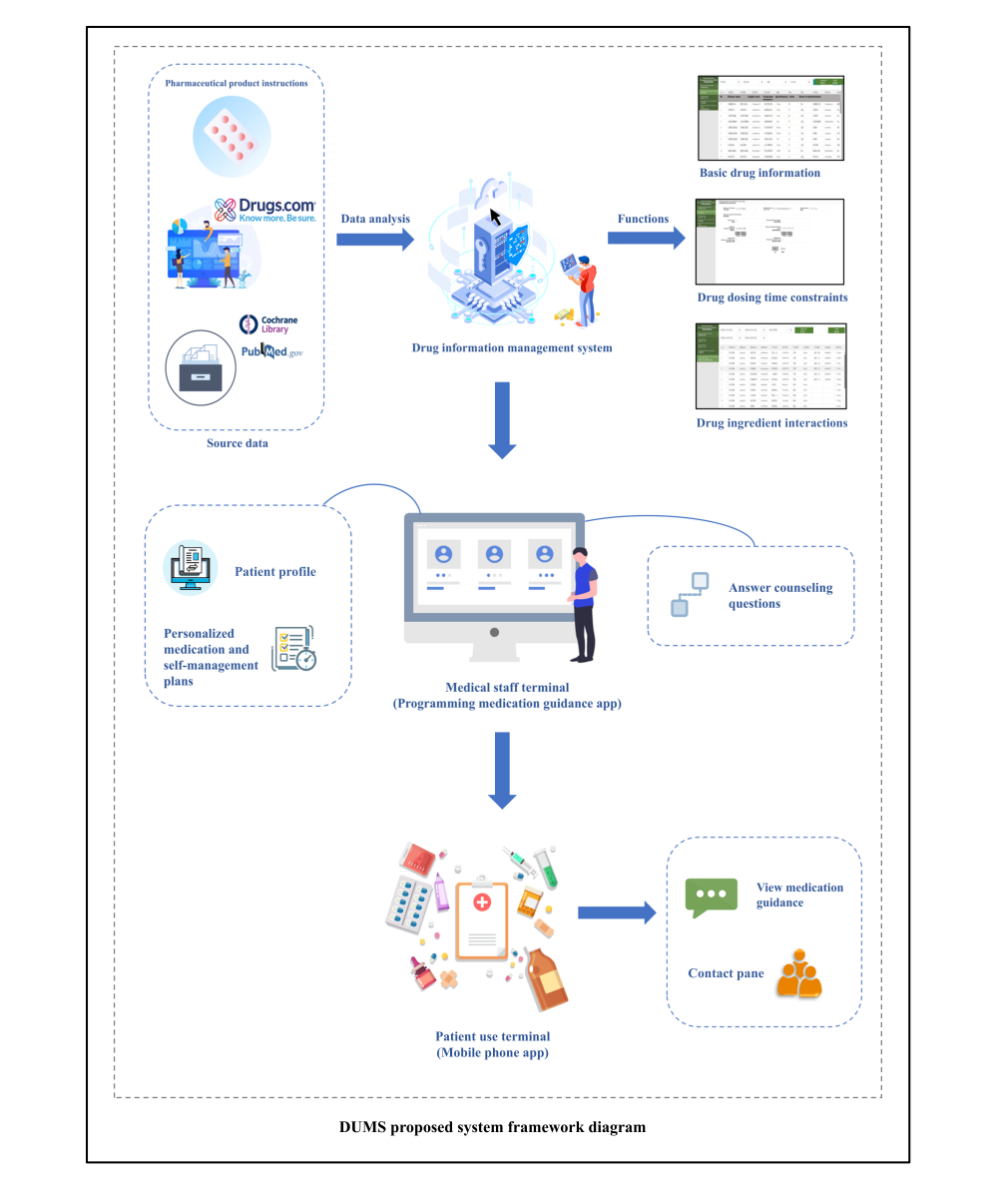
The architectural diagram and functional explanation of the Diabetes Universal Medication Schedule (DUMS) system.

Medication data were sourced from official medicine instructions and open-source medical professional websites, including information on ingredients, specifications, dosages, indications, and others. These data formed the foundational feature modules of the system. Dietary effects, interaction data, and physiologically based pharmacokinetic analysis were performed to establish pharmacological constraint information (Figure 1).

Interaction information was organized by drug active ingredient. Key data were extracted from official medicine description; the Drugs.com website; and scientific databases such as PubMed, Web of Science, and the Cochrane Library. A combination of subject-specific search terms (individual drug ingredients) and free-text queries was used to identify relevant studies on drug-dose relationships. Source data, including ingredient-ingredient expression of action, interaction severity, and treatment recommendations, were reviewed by 3 independent 5-member panels composing of physicians and pharmacists. The panels collaboratively assessed the data, ultimately confirming that the credibility of the data source and the logic of the interpretation.

#### Self-Management Program

Self-management is a dynamic process involving the identification of disease needs, activation of available resources, and adaptation to living with a chronic condition. Guidance and resolution from health care providers contribute to effective self-management. In addition to the regulating diet and exercise, a comprehensive management plan is developed based on the patient’s medication history, allergy history, adverse drug reaction history, and treatment adherence. Unstructured information is usually provided temporarily by health care professionals.

#### Reception and Interaction

The patient interface was built using the WeChat app framework featuring a responsive data binding system that synchronizes data with the user interface. This framework includes a view layer description language, a style language, and a JavaScript logic layer programming language. A mobile phone number serves as the user log-in ID to locate the medication data of the target patient.

### Dependability Tests

To evaluate the DUMS system’s real-world application performance, the prescription details of older patients with diabetes were extracted. After inputting the prescription details, the DUMS system extracts the established drug characteristics and constraints from the cloud-based drug database and does not adjust the output results (referred to as the “DUMS group”). Pharmacists then refined these preliminary results to create active output schemes that are better suited to clinical practice (hereinafter referred to as the “intervention group”). For comparison, the same prescription details were entered into ChatGPT-4, prompting it to generate medication schedules and self-management programs based on the drug label and relevant literature guidelines, where appropriate (hereafter referred to as the “GPT-4 group”; refer to Table S1 in Multimedia Appendix 1 for details).

Health care experts with professional titles at the associate senior level or above were invited to participate in the evaluation. They rated the results from the DUMS, intervention, and GPT-4 groups using a 5-point Likert scale following a horizontal comparison approach. Table 1 outlines the assessment indicators and their corresponding scoring criteria.

**Table 1:** Evaluation indicators and definitions.

Classification and indicators		Definitions
**Medication schedule**		
	Accuracy	Ensures that the use of medications aligns with mealtimes, drug-drug interactions, pharmacological timing, and daily routines
	Safety	When scheduling medication times, fully consider potential drug-drug interactions
	Adherence	Takes into account the convenience, rationality, and feasibility of medication
	Usefulness	It can effectively reduce the workload of health care workers and improve work efficiency
**Self-management program**		
	Accuracy	The content is accurate and error-free
	Comprehensiveness	Provides all necessary information for patients regarding medication, including usage precautions, identification and prevention of adverse reactions, and dietary therapy
	Richness	Provides information resources that exceed expectations
	Redundancy	Evaluates whether the content contains excessive, repetitive, or useless information; a score of 5 can be given if there is no excess or useless content
	Usefulness	Effectively reduces the burden on health care workers in education and public awareness efforts

### Statistical Analysis

SPSS (version 27.0; IBM Corp) was used to statistically analyze the data. Measurement information was expressed as mean (SD). Independent-sample ANOVA was used, followed by the least significant difference test for multiple comparison. The expert opinion harmonization coefficient (w) was calculated using Kendall W, with differences assessed through the chi-square (*χ*^2^) test. A *P*<.05 difference was considered statistically significant. Furthermore, the Bland-Altman method was used to generate scatter plots for pairwise comparisons of the 3 methods and to assess the degree of consistency.

### Ethical Considerations

This study was reviewed and approved by the Hospital Ethics Committee of Xiangya Hospital, Central South University (approval 2023030556). This study exclusively used prescribing information for test evaluation. There was no direct interaction with patients, in line with the principles of ethical conduct in the study. All data were deidentified to protect privacy and ensure confidentiality, with access restricted to authorized members of the research team.

## Results

### Construction of an Intelligent Medication Scheduling System

#### Enhancement of the DUMS System’s Cloud Database

From the endocrinology medication catalog, 475 commonly used medicines for patients with diabetes, including 146 imported medicines, were loaded. Each medicine’s information was summarized and organized into concise, comprehensive, and user-friendly entries based on the official instructions. Further, a total of 684 drug constraints were established for drugs, according to clinical recommendations related to chronopharmacology and diet-drug interactions.

After eliminating duplicate data, 8921 drug components were identified, and 12,351 drug interaction records were generated. Each interaction record included triple theoretical support: mechanism of action, interaction severity, and treatment recommendations. DDIs were classified into four categories based on severity: mild (n=6564, 53.15%), moderate (n=3392, 27.46%), severe (n=1876, 15.19%), and contraindicated (n=519, 4.2%). This classification helps medical professionals make quick and informed clinical decisions (refer to Table S2 in Multimedia Appendix 1 for details).

#### Individualized Medication Schedules and Self-Management Programs

**Figure 2:**
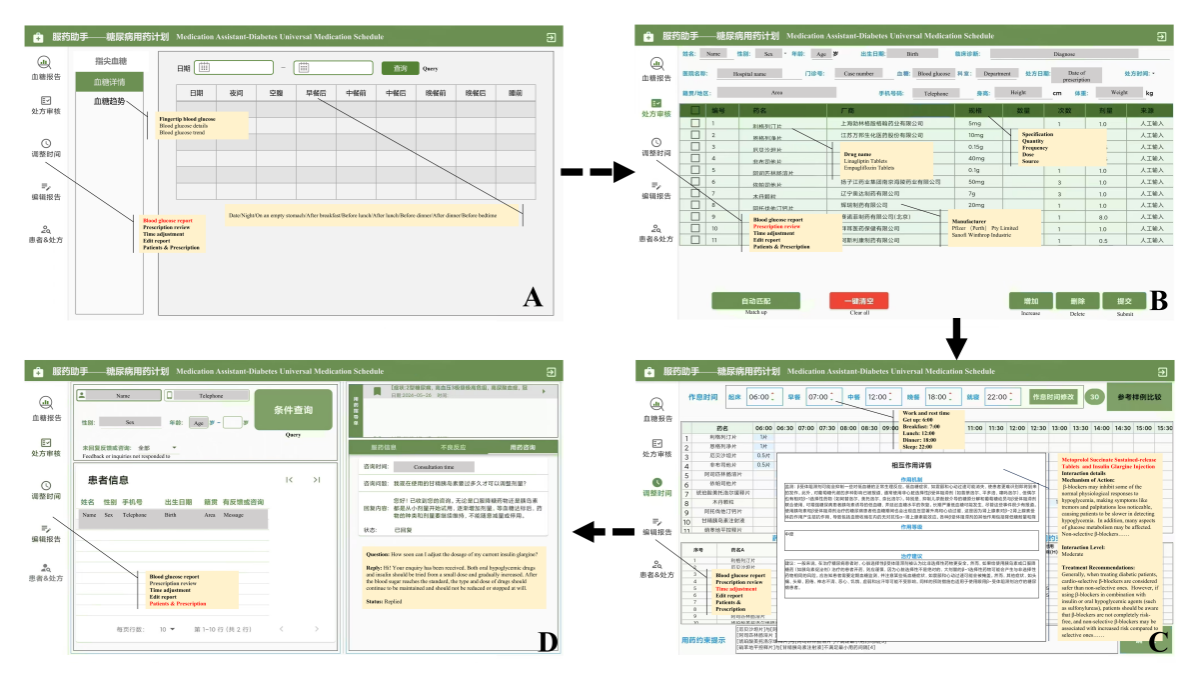
Diabetes Universal Medication Schedule (DUMS) interface: (A) blood glucose report; (B) prescription review; (C) time adjustment; (D) patients and prescription.

To facilitate timely management and personalized patient care, each patient’s profile was independently loaded with essential clinical information such as blood glucose status, medical diagnoses, and basic prescription. Default mealtimes were set at 7 AM, 12 PM, and 6 PM, with bedtime set at 10 PM, although health care providers can adjust these based on patient-specific needs and system-generated suggestions. Based on in-depth analysis of patients, providing more precise drug treatment plans and enhancing patient adherence can help improve long-term disease outcomes (Figure 2).

**Figure 3:**
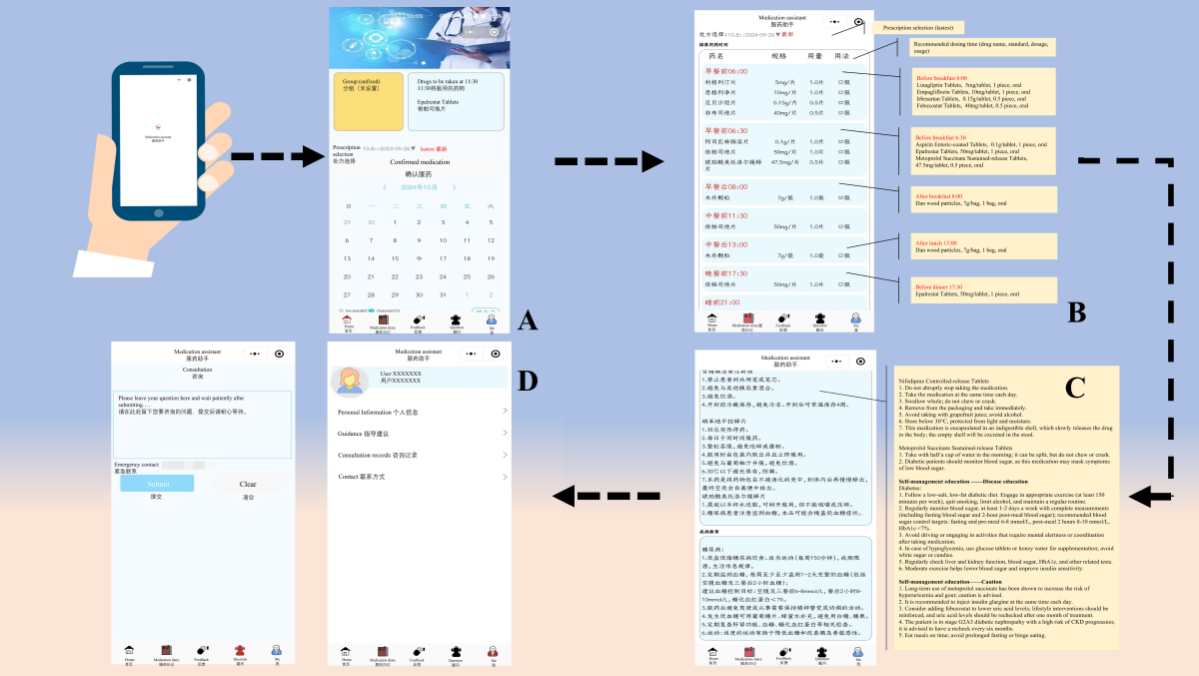
User terminal (medication assistant app): (A) time hint; (B) medication schedule; (C) self-management program; (D) consultation and settings.

Once the medication plan is arranged, patient can access detailed schedules given by the medical staff through the user terminal. For patients with diabetes, self-management programs cover exogenous insulin use (eg, insulin pens), awareness of drug side effects, dietary guidance, and prevention and control education. In addition, a communication module enables patients to submit inquiries regarding their current treatment status. Health care workers actively responded, reducing the likelihood of acute events while enhancing patients with diabetes’ understanding of and adherence to treatment plans (Figure 3).

### System Testing and Evaluation

A total of 12 specialists, comprising 8 general clinicians and 4 pharmacists, participated in this study, with a mean clinical experience of 3.08 (SD 0.99) years. The evaluation involved prescriptions for 20 older patients with diabetes, through 60 medication plans presented in 3 different output modes. Among the patients, more than half (n=14, 70%) were female, with an average medication frequency of 12.65 (SD 4.10) doses per day. The basic information about the patient cases is shown in Table 2.

**Table 2:** Patient and prescription information for the system test.

Characteristics		Value (N=20)
**Sex, n (%)**		
	Male	6 (30)
	Female	14 (70)
**Age (years), mean (SD)**		69.65 (4.17)
**Number of medications, n (%)**		
	<9	15 (75)
	≥9	5 (25)
**Number of chronic disease comorbidities, mean (SD)**		4.05 (2.04)
**Medication frequency (doses per day), mean (SD)**		12.65 (4.10)

**Figure 4:**
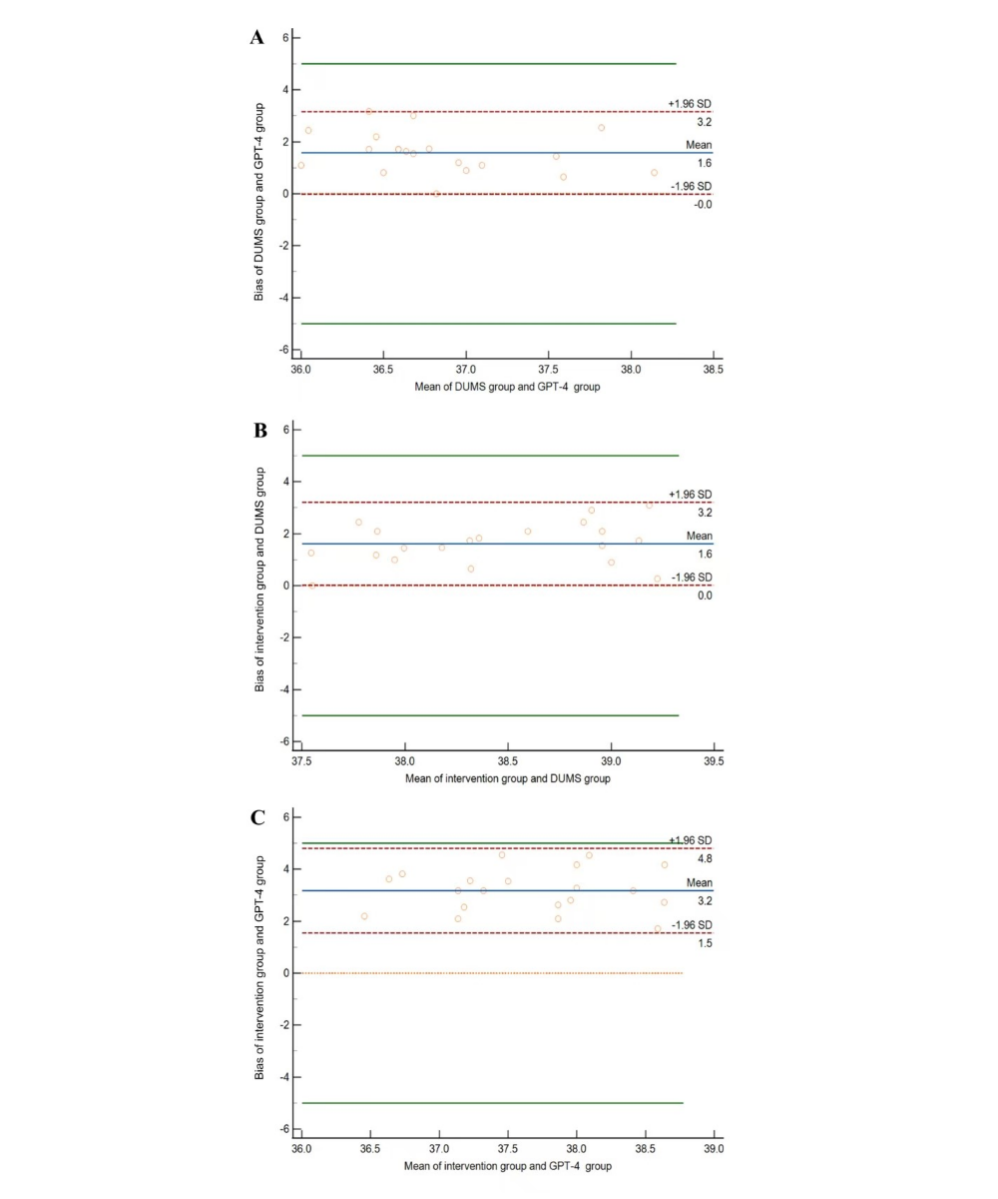
Bland-Altman plot for agreement analysis among the DUMS, intervention, and GPT-4 groups: (A) DUMS group vs GPT-4 group; (B) intervention group vs DUMS group; (C) intervention group vs GPT-4 group. Limits of agreement are shown as dashed, purplish red lines; bias is shown as solid blue lines; equivalence (difference=0) is shown as orange dashed lines; and maximum allowed difference between the groups is shown as solid green lines. DUMS: Diabetes Universal Medication Schedule.

The Kendall W coordination coefficient was used to calculate the agreement between raters (Kendall W for overall mark=0.21; *P*<.001), suggesting that the different scoring systems are reliable in producing reproducible results. Then, we supplemented the comparison between the two groups using the Bland-Altman method (Figure 4), revealing a mean bias between the DUMS group and the GPT-4 group as 1.57. The 95% CI for the lower limit of agreement (LoA) was –0.66 to 0.65, and the 95% CI of the upper LoA was 2.50-3.81. The mean bias between the intervention group and the DUMS group was 1.60, with 95% CI –0.65 to 0.67 for the lower LoA and 95% CI 2.54-3.86 for the upper LoA. No points of difference exceeded 95% LoAs. Comparing the intervention group to the GPT-4 group, the mean bias was 3.18. Most of the data points fell within the LoA, indicating no significant systematic bias or trend. Although some bias existed, the overall consistency was good.

The evaluation results revealed that the DUMS group achieved significantly higher accuracy and safety scores than the GPT-4 group (*P*<.001), with full consideration of multiple aspects of DDI, pharmacokinetics, and dietary restrictions. However, in order to reduce the occurrence of adverse events, the medication frequency was increased, which may reduce adherence to the medication plan. Regarding medication schedule usefulness, the DUMS group scored slightly higher than the GPT-4 group, and both DUMS and ChatGPT-4 were able to provide valuable information for pharmacists to assist patients in taking effective medication, but the difference was not statistically significant (*P*=.23). The self-management programs provided by DUMS not only deliver easy-to-understand instructions on medications but also offer multifaceted guidance on disease education, lifestyle, and dietary management, with positive feedback on accuracy, comprehensiveness, and richness (*P*<.001).

The total evaluation scores showed that the mean values of the DUMS group, intervention group, and GPT-4 group were 37.96 (SD 0.56), 39.52 (SD 0.80), and 36.35 (SD 0.89), respectively. The “adjusted” DUMS results (ie, the intervention group) were better than those of the other groups, with statistically significant differences (*P*<.001), as shown in Table 3.

**Table 3:** Results of the system testing and evaluation.

Classification and indicators		DUMS^a^ group, mean (SD)	Intervention group, mean (SD)	GPT-4 group, mean (SD)	*P* value (DUMS vs intervention)	*P* value (DUMS vs GPT-4)	*P* value (intervention vs GPT-4)
**Medication schedule**							
	Accuracy	4.43 (0.16)	4.49 (0.19)	3.84 (0.25)	.35	<.001	<.001
	Safety	4.44 (0.11)	4.44 (0.16)	3.91 (0.21)	.94	<.001	<.001
	Adherence	3.81 (0.34)	4.24 (0.24)	4.02 (0.25)	<.001	.03	.02
	Usefulness	4.11 (0.18)	4.30 (0.16)	4.04 (0.17)	.002	.23	<.001
**Self-management program**							
	Accuracy	4.56 (0.09)	4.64 (0.17)	4.34 (0.21)	.16	<.001	<.001
	Comprehensiveness	4.08 (0.21)	4.35 (0.15)	3.86 (0.18)	.04	<.001	<.001
	Richness	3.95 (0.26)	4.30 (0.24)	3.73 (0.19)	.03	<.001	<.001
	Redundancy	4.34 (0.17)	4.45 (0.19)	4.42 (0.19)	.07	.55	.61
	Usefulness	4.24 (0.12)	4.31 (0.13)	4.18 (0.13)	.16	.54	.04
**Total**		37.96 (0.56)	39.52 (0.80)	36.35 (0.89)	<.001	<.001	<.001

^a^DUMS: Diabetes Universal Medication Schedule.

## Discussion

### Principal Findings

Faced with the challenge of ensuring that patients with diabetes take the correct medication at the right time, the emergence of telemedicine and AI has provided researchers with new directions. In this study, we established constraint management of essential pharmaceutical products by summarizing the relationship between drugs and evidence-based arguments and created an individualized medication reminder platform.

Our system extends beyond traditional auxiliary models by offering a visually interactive personalized medication plan and a user-friendly interface. Through secondary data analysis, we establish a robust medicine constraint management and DDI risk assessment framework to guide health care providers in precision medication timing. A variety of complications in patients with diabetes often leads to complex medication management and puts patients at an increased risk of moderate to severe DDIs, which may threaten their lives or may deteriorate their quality of life [26,27]. To mitigate the above risks, a triple rationale for DDI is provided in our system, allowing health care providers to balance multiple drug demands with the possibility of harmful drug poisonings. Additionally, the system promotes DSMES by strengthening blood glucose management, ensuring patient take drugs correctly and on time, and developing exclusive planning [28].

ChatGPT is an evolving model in natural language processing, as Microsoft and OpenAI continue to develop it, and its potential applications in medical education have garnered a great deal of attention [29]. Previous studies indicate that ChatGPT-4 performs well in providing descriptive and objective content, often generating largely accurate information for a variety of medical queries [30-32]. In this study, to ensure consistency, the commands entered into ChatGPT-4 were manipulated by a single researcher, who entered the following instruction:

Patients (basic situation) and the prescription of the following drugs: (including drug name, dosage, frequency of use). It is assumed that the patient gets up at 6:00 every day, takes three meals at 7:00, 12:00 and 18:00 respectively, and goes to bed at 22:00. Please make a specific medication schedule and self-management plan for the patient according to the medicine instructions and relevant guidelines, and taking into account the patient's medication compliance.

However, the accuracy, comprehensiveness, and richness of the health education component of self-management provided by ChatGPT-4 could not replace that in the DUMS group and the “experienced” intervention group. A conceivable explanation is that, within the medical field, examinations often prioritize testing theoretical knowledge, frequently deviating from genuine patient-focused clinical environments. Such deviations might be more pronounced for LLMs, which are well versed in rapidly integrating available information. Another notable limitation is ChatGPT-4’s reliance on English-language training data, which affects its performance in other languages. The lack of culturally specific knowledge and the latest medical literature and data in other languages is a significant limitation of ChatGPT, which may lead to irrelevant or incorrect answers and conclusions [33,34]. Moreover, many scientific experts have pointed out that ChatGPT presents information redundantly and irrationally in most cases, offering repeated content and references, which may not be of obvious practical utility [35,36]. Although the development of machine learning and AI methods represents a significant technological advancement, and the output of ChatGPT-4 has a certain level of practicality, achieving reliable performance in various data scenarios still requires proper training of the model algorithms to maximize efficiency.

### Limitations

However, there are some limitations with the DUMS system. Its accuracy and usability rely heavily on the integrity of the drug information management database. Failure to update or expand the database promptly can result in suboptimal administration suggestions. Therefore, to continue expanding the number of drugs in the database and promptly improve the information resources, it is essential to develop a more efficient input mechanism for maintaining and managing the database, thus minimizing the risk of human errors or omissions. Additionally, comparing the performance of DUMS solely against that of ChatGPT-4 limits the generalizability of the findings to other intelligent systems or devices. Further validation is required to determine whether DUMS can perform consistently within its intended domain. Future iterations of DUMS will aim to enhance output quality by leveraging the imitative learning capabilities of LLMs, thereby improving the scientific and systematic nature of the output.

### Conclusion

The DUMS system represents an innovative and cost-effective mobile health solution. Without making any changes to the existing health care system, health care institutions can deploy our proposed system to provide safer and more rational medication services for patients with diabetes. The drug information and constraint management database, combined with the user-friendly interface developed in this study, supports health care professionals in creating accurate, personalized medication schedules and self-management programs. In addition to serving as a reminder, the inquiry component allows older individuals living independently to receive timely solutions when treatment issues arise. However, it remains unclear whether mobile health can help health care workers improve patient health literacy. Significant further research will be required from managers and end users to ensure the safety, reliability, and effectiveness of this technology.

## Appendix

Multimedia Appendix 1 Specific content of the expert evaluation and drug-drug interaction classification criteria.

## Abbreviations

AI: artificial intelligence
DDI: drug-drug interaction
DSMES: diabetes self-management education and support
DUMS: Diabetes Universal Medication Schedule
LLM: large language model
LoA: limit of agreement
USMLE: United States Medical Licensing Examination

## Data Availability

The datasets analyzed during this study are not publicly available to protect the privacy of individuals who participated in the study. Data are available from the corresponding author upon reasonable request.
