# Supplementary material for: Smart Pharmaceutical Monitoring System With Personalized Medication Schedules and Self-Management Programs for Patients With Diabetes: Development and Evaluation Study
Source: J Med Internet Res. 2025 Feb 11;27:e56737. doi: 10.2196/56737 (PMC11862767; doi:10.2196/56737)
Supplement: Multimedia Appendix 1 [file jmir_v27i1e56737_app1.docx]

**Supplemental Material**

**Table S1. Part of the specific content of expert evaluation.**

**Case 1——DUMS group**

| **Medication Guidance** | | | | | |
| --- | --- | --- | --- | --- | --- |
| Clinic Number: **Case 1** | | | Diagnosis：XXX | | |
| Name：XXX | | Sex：XXX | Age：XXX | | Date：XXX |
| **Medication schedule** | | | | | |
| **Recommended time** | **Medicine name** | | | **Norm** | **Usage and dosage** |
| Before breakfast 6:00 | Irbesartan Dispersible Tablets | | | 0.15g/tablet | 1 piece, oral |
|  | Dapagliflozin Tablets | | | 10mg/tablet | 1 piece, oral |
|  | Clopidogrel Bisulfate Tablets | | | 75mg/tablet | 1 piece, oral |
| Before breakfast 6:30 | Aspirin Enteric-coated Tablets | | | 0.1g/tablet | 1 piece, oral |
|  | Sodium Rabeprazole Enteric-coated Tablet | | | 20mg/tablet | 1 piece, oral |
| Breakfast time 7:00 | Pioglitazone Hydrochloride and Metformin Hydrochloride Tablets | | | 15mg:500mg  /tablet | 1 piece, oral |
|  | Acarbose Capsules | | | 50mg/pill | 2 pills, oral |
| Lunch time 12:00 | Acarbose Capsules | | | 50mg/pill | 2 pills, oral |
| Dinner time 18:00 | Pioglitazone Hydrochloride and Metformin Hydrochloride Tablets | | | 15mg:500mg  /tablet | 1 piece, oral |
|  | Acarbose Capsules | | | 50mg/pill | 2 pills, oral |
| Before bedtime 21:00 | Atorvastatin Calcium Tablets | | | 10mg/tablet | 2 pieces, oral |
| **Self-management program** | | | | | |
| **Drug description:**  **Aspirin Enteric-coated Tablets**   1. Take with an appropriate amount of water before meals. 2. Do not drink alcohol during the use of this medication. 3. If you experience discomfort or mild stomach pain, please consult a pharmacist.   **Rabeprazole Sodium Enteric-coated Tablets**   1. Swallow the whole tablet 30 minutes before breakfast. Do not chew or crush the tablet. 2. Avoid alcohol and smoking during treatment to prevent reduced efficacy.   **Pioglitazone and Metformin Tablets**   1. Take with meals. 2. Avoid alcohol. 3. If gastrointestinal side effects occur, consider taking the medication after meals.   **Acarbose Capsules**   1. Swallow the whole capsule immediately before meals.   **Dapagliflozin Tablets**   1. Ensure adequate daily hydration (1500-2000 ml, approximately 4-5 bottles of mineral water). 2. Maintain proper foot hygiene and care. Seek medical attention promptly if foot ulcers or infections occur.   **Clopidogrel Bisulfate Tablets**   1. Take the medication at the same time every day. 2. Can be taken with or without food. If gastrointestinal discomfort occurs, it can be taken with food   **Atorvastatin Calcium Tablets**   1. Avoid excessive alcohol consumption and the intake of grapefruit or grapefruit juice during treatment. 2. Diabetic patients should monitor their blood sugar levels.   **Disease Management:**   1. Engage in appropriate physical activity (at least 150 minutes per week), quit smoking, limit alcohol consumption, and maintain a regular daily routine. 2. Regularly monitor blood glucose levels, at least 1-2 days per week, including both fasting blood glucose and 2-hour postprandial blood glucose. 3. Avoid driving or engaging in activities that require mental alertness or coordination after taking medication. 4. In case of hypoglycemia, use glucose tablets or honey water for supplementation, and avoid using white sugar or candies. 5. Regularly monitor liver and kidney function, blood glucose, HbA1c, and other related tests. | | | | | |

**Table S1. Part of the specific content of expert evaluation (continued).**

**Case 1——Intervention group**

| **Medication Guidance** | | | | | |
| --- | --- | --- | --- | --- | --- |
| Clinic Number: **Case 1** | | | Diagnosis：XXX | | |
| Name：XXX | | Sex：XXX | Age：XXX | | Date：XXX |
| **Medication schedule** | | | | | |
| **Recommended time** | **Medicine name** | | | **Norm** | **Usage and dosage** |
| Before breakfast 6:30 | Aspirin Enteric-coated Tablets | | | 0.1g/tablet | 1 piece, oral |
|  | Irbesartan Dispersible Tablets | | | 0.15g/tablet | 1 piece, oral |
|  | Sodium Rabeprazole Enteric-coated Tablet | | | 20mg/tablet | 1 piece, oral |
| Breakfast time 7:00 | Pioglitazone Hydrochloride and Metformin Hydrochloride Tablets | | | 15mg:500mg  /tablet | 1 piece, oral |
|  | Acarbose Capsules | | | 50mg/pill | 2 pills, oral |
|  | Dapagliflozin Tablets | | | 10mg/tablet | 1 piece, oral |
| Lunch time 12:00 | Acarbose Capsules | | | 50mg/pill | 2 pills, oral |
| Dinner time 18:00 | Pioglitazone Hydrochloride and Metformin Hydrochloride Tablets | | | 15mg:500mg  /tablet | 1 piece, oral |
|  | Acarbose Capsules | | | 50mg/pill | 2 pills, oral |
| Before bedtime 21:00 | Clopidogrel Bisulfate Tablets | | | 75mg/tablet | 1 piece, oral |
|  | Atorvastatin Calcium Tablets | | | 10mg/tablet | 2 pieces, oral |
| **Self-management program** | | | | | |
| **Drug description:**  **Aspirin Enteric-coated Tablets**   1. Take with an appropriate amount of water before meals. 2. Do not drink alcohol during the use of this medication. 3. If you experience discomfort or mild stomach pain, please consult a pharmacist. 4. If you are currently using aspirin or planning to take any non-steroidal anti-inflammatory drugs (NSAIDs), you should consult a doctor or pharmacist.   **Rabeprazole Sodium Enteric-coated Tablets**   1. Swallow the whole tablet 30 minutes before breakfast. Do not chew or crush the tablet. 2. Avoid alcohol and smoking during treatment to prevent reduced efficacy.   **Pioglitazone and Metformin Tablets**   1. Take with meals. 2. Avoid alcohol. 3. If gastrointestinal side effects occur, consider taking the medication after meals. 4. If you experience symptoms such as shortness of breath, severe fatigue, chest pain, or persistent gastrointestinal discomfort, you should stop the medication immediately and consult a doctor. 5. Maintain a healthy diet and engage in moderate exercise to control weight and blood glucose levels. 6. Avoid excessive fatigue and maintain a regular daily routine.   **Acarbose Capsules**   1. Swallow the whole capsule immediately before meals. 2. Avoid excessive intake of carbohydrates, especially foods with high sugar content after meals.   **Dapagliflozin Tablets**   1. Ensure adequate daily hydration (1500-2000 ml, approximately 4-5 bottles of mineral water). 2. Maintain proper foot hygiene and care. Seek medical attention promptly if foot ulcers or infections occur.   **Clopidogrel Bisulfate Tablets**   1. Take the medication at the same time every day. 2. Can be taken with or without food. If gastrointestinal discomfort occurs, it can be taken with food 3. If you need to undergo surgery or dental treatment, be sure to inform the doctor that you are using clopidogrel, as the medication can affect blood clotting and may need to be stopped before the procedure. 4. Regular monitoring: While using clopidogrel, it is important to have regular blood tests, especially platelet count, to ensure the safety of the medication.   **Atorvastatin Calcium Tablets**   1. Avoid excessive alcohol consumption and the intake of grapefruit or grapefruit juice during treatment. 2. Diabetic patients should monitor their blood sugar levels.   **Disease Management:**   1. Follow a low-salt, low-fat diabetic diet. Prioritize high-fiber foods such as whole grains, legumes, and vegetables, and control the carbohydrate intake at each meal. Include more fiber-rich foods, such as vegetables, legumes, and whole grains, in your diet. 2. Engage in appropriate physical activity (at least 150 minutes per week), quit smoking, limit alcohol consumption, and maintain a regular daily routine. 3. Regularly monitor blood glucose levels, at least 1-2 days per week, including both fasting blood glucose and 2-hour postprandial blood glucose. 4. Avoid driving or engaging in activities that require mental alertness or coordination after taking medication. 5. In case of hypoglycemia, use glucose tablets or honey water for supplementation, and avoid using white sugar or candies. 6. Regularly monitor liver and kidney function, blood glucose, HbA1c, and other related tests. | | | | | |

**Table S1. Part of the specific content of expert evaluation (continued).**

**Case 1——GPT-4 group**

ChatGPT4 generates medication guidance in no fixed format, so we standardized the format of its output before expert review but the content remained unchanged.

| **Medication Guidance** | |
| --- | --- |
| Clinic Number: **Case 1** | |
| Name：XXX | Diagnosis：XXX |
| Medication Situation：XXX | |
| **Aspirin Enteric-coated Tablets (0.1g, qd)**   - **Dosage time**: It is recommended to take it after breakfast, around 7:00 AM. - **How to take**: Swallow the whole tablet, do not chew. - **Precautions**: - Long-term use of aspirin may cause gastrointestinal discomfort or bleeding, so it is important to monitor gastrointestinal symptoms. - If black stools or significant abdominal pain occur, seek medical attention immediately. - Do not stop or adjust the dosage without consulting your doctor. | |
| **Irbesartan Dispersible Tablets (0.15g, qd)**   **Dosage time**: It is recommended to take it in the morning at 7:00 AM together with aspirin.   **How to take**: The tablet can be swallowed directly or dispersed in a small amount of water before taking.   **Precautions**:   - Regularly monitor blood pressure to ensure the medication is effective. - If symptoms of hypotension occur (e.g., dizziness, fatigue), consult your doctor. - When used in combination with certain medications (such as diuretics or potassium supplements), monitoring electrolyte levels may be necessary. | |
| **Sodium Rabeprazole Enteric-coated Tablet** **(20mg, qd)**  ** Dosage time:** It is recommended to take it after waking up at 6:00 AM, on an empty stomach.  ** How to take:** Swallow the whole tablet, do not chew.  ** Precautions:**   - Take on an empty stomach to ensure optimal absorption. - Do not take with other medications, especially supplements containing calcium, iron, or magnesium, as they may affect drug absorption. | |
| **Acarbose Capsules (50mg, tid)**   - **Dosage time:** Take 30 minutes before each meal (6:30 AM, 11:30 AM, 5:30 PM). - **How to take:** Take with food before each meal. - **Precautions:** - This medication is used to control postprandial blood sugar, so it must be taken before meals. - Acarbose may cause bloating, diarrhea, or other gastrointestinal discomfort. If symptoms are severe, consult your doctor. | |
| **Pioglitazone and Metformin Tablets (15mg:500mg, bid)**   - **Dosage time:** Take one tablet in the morning and one in the evening, recommended at 7:00 AM and 6:00 PM after dinner. - **How to take:** Swallow the whole tablet, do not chew. - **Precautions:** - This medication is primarily used to improve diabetes control. Follow the doctor's instructions. - If symptoms like weight gain, edema, or shortness of breath occur, seek medical attention immediately. | |
| **Dapagliflozin Tablets (10mg, qd)**   - **Dosage time:** It is recommended to take it in the morning at 7:00 AM, along with Pioglitazone and Metformin Tablets. - **How to take:** Swallow the whole tablet, do not chew. - **Precautions:** - This medication helps control blood sugar and has a diuretic effect, so be sure to stay adequately hydrated. - If symptoms of urinary tract infection or genital infections occur, seek medical attention immediately. | |
| **Clopidogrel Sulfate Tablets (75mg, qd)**   - **Dosage time:** It is recommended to take it with breakfast. - **How to take:** Swallow the whole tablet, do not chew. - **Precautions:** - This medication has antiplatelet effects and reduces blood clot formation. - Avoid taking it with other medications that affect coagulation, especially nonsteroidal anti-inflammatory drugs (NSAIDs) or other anticoagulants. - Monitor for signs of bleeding, such as bruising, gum bleeding, etc. If you experience any discomfort, contact your doctor promptly. | |
| **Atorvastatin Calcium Tablets (20mg, qd)**   - **Dosage time:** It is recommended to take it at 10:00 PM. - **How to take:** Swallow the whole tablet, do not chew. - **Precautions:** - Avoid heavy drinking to reduce the risk of muscle damage. - Regularly check liver function, and inform your doctor if you experience muscle pain, fatigue, or any discomfort. | |
| **Additional recommendations**   - Maintain a healthy diet, control sugar intake, and engage in appropriate physical activity. - Regular follow-ups to monitor treatment outcomes and any adverse reactions, and adjust the treatment plan as necessary. | |

| **Table S1. Part of the specific content of expert evaluation (continued).**  **Case 2——DUMS group** | | | | | |
| --- | --- | --- | --- | --- | --- |
| **Medication Guidance** | | | | | |
| Clinic Number: **Case 2** | | | Diagnosis：XXX | | |
| Name：XXX | | Sex：XXX | Age：XXX | | Date：XXX |
| **Medication schedule** | | | | | |
| **Recommended time** | **Medicine name** | | | **Norm** | **Usage and dosage** |
| Before breakfast 6:30 | Thioctic Acid Tablets | | | 0.3g/tablet | 2 pieces, oral |
|  | Epalrestat Tablets | | | 50mg/tablet | 1 piece, oral |
|  | Nifedipine Controlled-release Tablets | | | 30mg/tablet | 1 piece, oral |
| Breakfast time 7:00 | Insulin Aspart 30 Injection | | | 300/iu | 30iu |
|  | Pioglitazone Hydrochloride and Metformin Hydrochloride Tablets | | | 50mg/tablet | 1 piece, oral |
|  | Miglitol Tablets | | | 50mg/tablet | 1 piece, oral |
| After Breakfast 8:00 | Indobufen Tablets | | | 0.2g/tablet | 1/2 piece, oral |
|  | Beraprost Sodium Tablets | | | 40μg/tablet | 1 piece, oral |
| Before lunch 11:30 | Epalrestat Tablets | | | 50mg/tablet | 1 piece, oral |
| Lunch time 12:00 | Miglitol Tablets | | | 50mg/tablet | 1 piece, oral |
| After lunch 13:00 | Beraprost Sodium Tablets | | | 40μg/tablet | 1 piece, oral |
| Before dinner 17:30 | Epalrestat Tablets | | | 50mg/tablet | 1 piece, oral |
| Dinner time 18:00 | Insulin Aspart 30 Injection | | | 300/iu | 30iu |
|  | Pioglitazone Hydrochloride and Metformin Hydrochloride Tablets | | | 15mg:500mg  /tablet | 1 piece, oral |
|  | Miglitol Tablets | | | 50mg/tablet | 1 piece, oral |
| After dinner 19:00 | Indobufen Tablets | | | 0.2g/tablet | 1/2 piece, oral |
|  | Beraprost Sodium Tablets | | | 40μg/tablet | 1 piece, oral |
| **Self-management program** | | | | | |
| **Drug description:**  **Insulin Aspart 30 Injection**   1. Avoid mixing with other insulins. 2. Do not shake. 3. Store unopened vials at 2-8℃ (refrigeration); avoid freezing. After opening, store at room temperature (10-30℃) for 4 weeks. 4. This product is administered via subcutaneous injection, with injection sites including the thigh or abdominal wall. If convenient, the buttocks or deltoid area may also be used. Injection points should be rotated within the same injection area to reduce the risk of lipodystrophy. 5. Take extreme care when handling used needles to avoid accidental injury. 6. Do not share injection pens or needles with others, as this may cause cross-infection. 7. Keep injection pens and needles out of reach of others, especially children.   **Pioglitazone and Metformin Tablets**   1. Take with meals. 2. Avoid alcohol. 3. If gastrointestinal side effects occur, consider taking the medication after meals. 4. Consult a doctor or pharmacist if medications cause gastrointestinal side effects, such as nausea, bloating, or diarrhea.   **Miglitol Tablets**   1. If hypoglycemia occurs when using this medicine, do not take supplements such as sucrose, candy or household sugar, but take fructose or glucose.   **Thioctic Acid Tablets**   1. Take it separately from food 2. Long-term alcohol use may affect the success rate of treatment, so it is recommended to abstain from alcohol as much as possible, as well as during periods without treatment.   **Epalrestat Tablets**   1. Take before meals. 2. During medication, urine may appear reddish brown, which is normal   **Nifedipine Controlled-release Tablets**   1. Do not stop the medication suddenly. 2. Take the medication at the same time every day. 3. Swallow the tablet whole; do not chew or crush it. 4. Remove the medication from its packaging and take it immediately. 5. Avoid taking it with grapefruit juice and avoid alcohol. 6. Store below 30℃, protect from light, and keep dry.   **Indobufen Tablets**   1. Use with caution in patients with active gastrointestinal lesions and those taking nonsteroidal anti-inflammatory drugs (NSAIDs). 2. Avoid concurrent use with other anticoagulants or aspirin.   **Beraprost Sodium Tablets**   1. Take after meals. 2. Patients who are using anticoagulants, antiplatelet drugs, or thrombolytics are advised to consult a doctor or pharmacist before taking the medication.   **Disease Management:**   1. Engage in appropriate physical activity (at least 150 minutes per week), quit smoking, limit alcohol consumption, and maintain a regular daily routine. 2. Regularly monitor blood glucose levels, at least 1-2 days per week, including both fasting blood glucose and 2-hour postprandial blood glucose. 3. Avoid driving or engaging in activities that require mental alertness or coordination after taking medication. 4. In case of hypoglycemia, use glucose tablets or honey water for supplementation, and avoid using white sugar or candies. 5. Regularly monitor liver and kidney function, blood glucose, HbA1c, and other related tests. | | | | | |

| **Table S1. Part of the specific content of expert evaluation (continued).**  **Case 2——Intervention group** | | | | | |
| --- | --- | --- | --- | --- | --- |
| **Medication Guidance** | | | | | |
| Clinic Number: **Case 2** | | | Diagnosis：XXX | | |
| Name：XXX | | Sex：XXX | Age：XXX | | Date：XXX |
| **Medication schedule** | | | | | |
| **Recommended time** | **Medicine name** | | | **Norm** | **Usage and dosage** |
| Before breakfast 6:30 | Thioctic Acid Tablets | | | 0.3g/tablet | 2 pieces, oral |
|  | Epalrestat Tablets | | | 50mg/tablet | 1 piece, oral |
|  | Nifedipine Controlled-release Tablets | | | 30mg/tablet | 1 piece, oral |
|  | Insulin Aspart 30 Injection | | | 300/iu | 30iu |
|  | Miglitol Tablets | | | 50mg/tablet | 1 piece, oral |
| After Breakfast 8:00 | Pioglitazone Hydrochloride and Metformin Hydrochloride Tablets | | | 15mg:500mg  /tablet | 1 piece, oral |
|  | Indobufen Tablets | | | 0.2g/tablet | 1/2 piece, oral |
|  | Beraprost Sodium Tablets | | | 40μg/tablet | 1 piece, oral |
| Before lunch 11:30 | Epalrestat Tablets | | | 50mg/tablet | 1 piece, oral |
|  | Miglitol Tablets | | | 50mg/tablet | 1 piece, oral |
| After lunch 13:00 | Beraprost Sodium Tablets | | | 40μg/tablet | 1 piece, oral |
| Before dinner 17:30 | Epalrestat Tablets | | | 50mg/tablet | 1 piece, oral |
|  | Insulin Aspart 30 Injection | | | 300/iu | 30iu |
|  | Miglitol Tablets | | | 50mg/tablet | 1 piece, oral |
| After dinner 19:00 | Pioglitazone Hydrochloride and Metformin Hydrochloride Tablets | | | 15mg:500mg | 1 piece, oral |
|  | Indobufen Tablets | | | 0.2g/tablet | 1/2 piece, oral |
|  | Beraprost Sodium Tablets | | | 40μg/tablet | 1 piece, oral |
| **Self-management program** | | | | | |
| **Drug description:**  **Insulin Aspart 30 Injection**   1. Avoid mixing with other insulins. 2. Do not shake. 3. Store unopened vials at 2-8℃ (refrigeration); avoid freezing. After opening, store at room temperature (10-30℃) for 4 weeks. 4. This product is administered via subcutaneous injection, with injection sites including the thigh or abdominal wall. If convenient, the buttocks or deltoid area may also be used. Injection points should be rotated within the same injection area to reduce the risk of lipodystrophy. 5. Take extreme care when handling used needles to avoid accidental injury. 6. Do not share injection pens or needles with others, as this may cause cross-infection. 7. Keep injection pens and needles out of reach of others, especially children.   **Pioglitazone and Metformin Tablets**   1. Take with meals. 2. Avoid alcohol. 3. If gastrointestinal side effects occur, consider taking the medication after meals.   **Miglitol Tablets**   1. If hypoglycemia occurs when using this medicine, do not take supplements such as sucrose, candy or household sugar, but take fructose or glucose. 2. During the medication period, it is very important to follow dietary control and engage in appropriate exercise.   **Thioctic Acid Tablets**   1. Take it separately from food 2. Long-term alcohol use may affect the success rate of treatment, so it is recommended to abstain from alcohol as much as possible, as well as during periods without treatment.   **Epalrestat Tablets**   1. Take before meals. 2. During medication, urine may appear reddish brown, which is normal   **Nifedipine Controlled-release Tablets**   1. Do not stop the medication suddenly. 2. Take the medication at the same time every day. 3. Swallow the tablet whole; do not chew or crush it. 4. Remove the medication from its packaging and take it immediately. 5. Avoid taking it with grapefruit juice and avoid alcohol. 6. Store below 30℃, protect from light, and keep dry.   **Indobufen Tablets**   1. Use with caution in patients with active gastrointestinal lesions and those taking nonsteroidal anti-inflammatory drugs (NSAIDs). 2. Avoid concurrent use with other anticoagulants or aspirin.   **Beraprost Sodium Tablets**   1. Take after meals. 2. Patients who are using anticoagulants, antiplatelet drugs, or thrombolytics are advised to consult a doctor or pharmacist before taking the medication. 3. After using this product, caution is needed when driving or operating dangerous machinery.   **Disease Management:**   1. Eat small, frequent meals instead of large meals to reduce gastrointestinal discomfort. 2. Focus on easily digestible foods, such as low-fiber, soft, and low-fat options. 3. Avoid spicy, greasy, and acidic foods that may worsen gastrointestinal symptoms. 4. Engage in appropriate physical activity (at least 150 minutes per week), quit smoking, limit alcohol consumption, and maintain a regular daily routine. 5. Regularly monitor blood glucose levels, at least 1-2 days per week, including both fasting blood glucose and 2-hour postprandial blood glucose. 6. Avoid driving or engaging in activities that require mental alertness or coordination after taking medication. 7. In case of hypoglycemia, use glucose tablets or honey water for supplementation, and avoid using white sugar or candies. 8. Regularly monitor liver and kidney function, blood glucose, HbA1c, and other related tests. | | | | | |

| **Table S1. Part of the specific content of expert evaluation (continued).**  **Case 2——GPT-4 group** | |
| --- | --- |
| **Medication Guidance** | |
| Clinic Number: **Case 2** | |
| Name：XXX | Diagnosis：XXX |
| Medication Situation：XXX | |
| **Insulin Aspart 30 Injection (30iu, bid)**   - **Dosage time**: It is recommended to take it before meals, around 7:00 AM, 12:00AM. - **How to take**: This medication is for subcutaneous injection only. - **Precautions**: - Check the transparency and color of the insulin injection, do not use if there is precipitation or discoloration. Transposition during injection to avoid induration. - Keep clean and hygienic when using, and the injection site should be changed alternately to prevent skin induration. | |
| **Miglitol Tablets (50mg, tid)**   **Dosage time**: It is recommended to take it before meals, around 7:00 AM, 12:00AM, 6:00PM.   **How to take**: Need to be taken in the dining together with the first bite of food to chew.   **Precautions**:   - Hypoglycemia caused by miglitol cannot be treated with sucrose or regular candies; glucose tablets or glucose water must be used instead. - Symptoms of hypoglycemia include dizziness, palpitations, and sweating, which should be taken seriously and addressed promptly. | |
| **Thioctic Acid Tablets** **(0.6g, qd)**  ** Dosage time:** It is recommended to take it after waking up at 6:00 AM, on an empty stomach.  ** How to take:** Swallow the whole tablet, do not chew.  ** Precautions:**   - Hypoglycemia caused by miglitol cannot be treated with sucrose or regular candies; glucose tablets or glucose water must be used instead. | |
| **Epalrestat Tablets (50mg, tid)**   - **Dosage time:** Take 30 minutes before each meal (7:00 AM, 12:00 AM, 18:00 PM). - **How to take:**  Swallow the whole tablet, do not chew. - **Precautions:** - A low-cholesterol diet may be required during medication use. - After taking this product, the urine may appear brown red, so some test items may be affected. | |
| **Pioglitazone and Metformin Tablets (15mg:500mg, bid)**   - **Dosage time:** It is recommended to take it before meals, around 7:00 AM, 12:00AM. - **How to take:** Swallow the whole tablet, do not chew. - **Precautions:** - This medication is primarily used to improve diabetes control. Follow the doctor's instructions. - If symptoms like weight gain, edema, or shortness of breath occur, seek medical attention immediately. | |
| **Nifedipine Controlled-release Tablets (30mg, qd)**   - **Dosage time:** It is recommended to take before bedtime 10:00 PM. - **How to take:** Swallow the whole tablet, do not chew. - **Precautions:** - It is recommended to take it at the same time every day to keep the blood concentration stable. - Avoid taking this medication with grapefruit juice, as it may affect drug metabolism, leading to enhanced efficacy or increased side effects. - Monitor blood pressure changes during use and avoid standing up suddenly or changing positions too quickly to prevent orthostatic hypotension and fainting. - Abrupt discontinuation of the medication should be avoided during treatment to prevent blood pressure rebound or worsening angina. | |
| **Indobufen Tablets (0.1g, bid)**   - **Dosage time:** It is recommended to take it after meals, around 7:00 AM, 12:00AM. - **How to take:** Do not chew. - **Precautions:** - If abnormal bleeding occurs during medication use (such as gum bleeding, nosebleeds, or blood in the urine), stop the medication immediately and consult a doctor. - Avoid alcohol consumption, as it may exacerbate gastrointestinal irritation or increase the risk of bleeding. - Inform your doctor before surgery if you are taking indobufen, as the medication may need to be discontinued to reduce the risk of intraoperative bleeding. | |
| **Beraprost Sodium Tablets (40μg, tid)**   - **Dosage time:** It is recommended to take with a meal or dinner - **How to take:** Swallow the whole tablet, do not chew. - **Precautions:** - Do not arbitrarily increase or decrease the dosage, and do not discontinue the medication without medical advice. - The medication may cause a drop in blood pressure during use. Patients should change positions slowly and avoid standing up suddenly to prevent dizziness or fainting. | |
| **Additional recommendations:**   - Maintain a healthy diet, control sugar intake, and engage in appropriate physical activity. - Regular follow-ups to monitor treatment outcomes and any adverse reactions, and adjust the treatment plan as necessary. | |

**Table S2. DDI classification criteria.**

| **Degree** | **Definition** | **Characteristics** |
| --- | --- | --- |
| Mild | This type of interaction has little to no impact on the clinical effects for the patient and typically does not require any special treatment intervention. | 1. Minimal impact on efficacy or side effects. 2. No need to adjust drug dosage or discontinue use. 3. Monitoring or attention is suggested. 4. Does not affect treatment plan. |
| Moderate | This type of interaction may affect the drug's efficacy or side effects, and may require dosage adjustment or treatment plan changes. | 1. May impact drug efficacy or increase the risk of side effects. 2. Dosage adjustments or treatment changes may be required. 3. Requires attention from a healthcare provider. |
| Severe | This type of interaction can pose a serious threat to the patient’s health, possibly requiring adjustments or discontinuation of medication and may even be life-threatening. | 1. Significantly impacts efficacy, potentially leading to serious side effects or life-threatening risks. 2. Immediate adjustments or discontinuation of treatment may be necessary. 3. Strongly advised to avoid this combination. |
| Contraindicated | This interaction should never occur under any circumstances and is considered an absolute contraindication. The drug combination may pose a fatal risk to the patient's health and could lead to severe health damage | 1. May lead to fatal or extremely severe side effects. 2. Absolutely not recommended, must avoid the drug combination. 3. Requires discontinuation of the drug or replacement of the treatment plan. |
